# Die ventrale Abstützung bei dorsalen lumbalen Fusionsoperationen: Ein Fallbericht mit vielen Zwischenfällen

**DOI:** 10.1007/s00132-021-04140-6

**Published:** 2021-08-17

**Authors:** Moritz Mederake, Christian Walter

**Affiliations:** Orthopädische Universitätsklinik Tübingen, Hoppe-Seyler-Str. 3, 72076 Tübingen, Deutschland

**Keywords:** Lumbalregion, Wirbelkörperfusion, Wirbelsäule, Spondylodiszitis, Spondylolisthese, Lumbar region, Spinal Fusion, Spine, Spondylodiscitis, Spondylolisthesis

## Abstract

Die ventrale Abstützung im Rahmen dorsaler Fusionsoperationen ist insbesondere bei instabilen Pathologien, wie Spondylolisthesen oder Spondylodiszitiden, entscheidend für das Operationsergebnis. Der komplikative Verlauf einer Patientin mit simultan bestehender Spondylolisthese und Spondylodiszitis wird dargestellt und anhand der Literatur und des eigenen Behandlungsalgorithmus reevaluiert. Bei alleiniger Spondylodiszitis ist ein Beckenkamminterponat als Abstützung ausreichend. Bei zusätzlichen Störungen des sagittalen Profils ist eine Cage-Implantation zu bevorzugen.

## Abstract (en)

Anterior column support in posterior lumbar fusion surgery is essential for the outcome of the operation, particularly in the case of unstable pathologies such as spondylolisthesis or spondylodiscitis. The complicated case of a patient with simultaneous spondylolisthesis and spondylodiscitis is presented and reevaluated based on the literature and our own treatment algorithm. In the case of spondylodiscitis alone, iliac crest interposal is sufficient as a support. In the case of additional disturbances of the sagittal profile, a cage implantation is preferred.

## Anamnese

**Figure 1:**
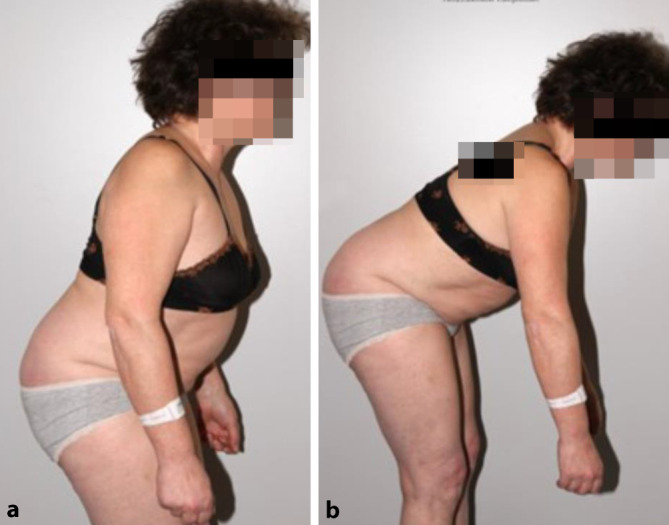

Die 51-jährige Patientin klagte über ausgeprägte Lumbofemoralgien beidseits mit Hypästhesien an beiden ventralen Oberschenkeln. Zudem beeinträchtigte sie der massive Lotüberhang des Oberkörpers (Abb. 1). Nebenbefundlich besteht eine chronische Hepatitis-C-Infektion und Methadon-Substitution bei Z. n. i.v.-Drogenabusus.

**Figure 2:**
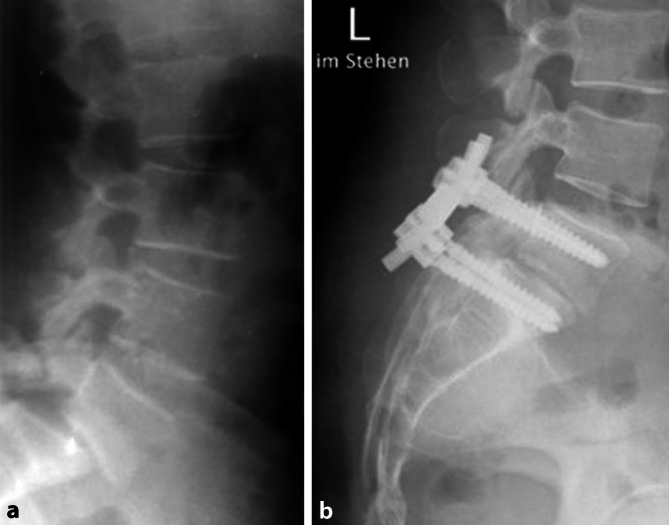

Als Ausgangssituation bestand 9 Jahre zuvor eine Spondylodiszitis bei Spondylolisthesis vera LWK5/SWK1 Meyerding Typ II, welche zunächst mit einer dorsalen Spondylodese und Bandscheibenresektion ohne ventrale Abstützung versorgt wurde (Abb. 2).

**Figure 3:**
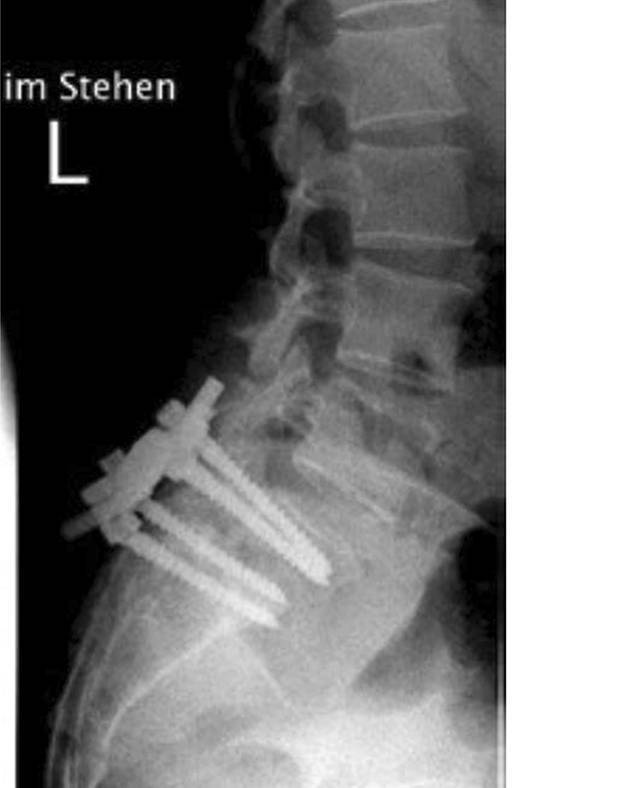

Bei weiter bestehenden Infektzeichen waren weitere Revisionen, u. a. eine Beckenkammspongiosainterposition am 14. postoperativen Tag, erforderlich. Allerdings fand sich bereits nach einem Monat eine Schraubenlockerung mit progredienter Einschmelzung des LWK5 und lokaler Kyphosierung (Abb. 3), welche zu einer Verlängerung bis LWK4 mit ventraler trikortikaler Spantransplantation vom Beckenkamm 3 Monate nach der Primäroperation führte.

Trotz multipler weiterer Eingriffe (Tab. 1) zeigte sich in den folgenden 5 Jahren eine sagittale Imbalance mit progredienter Lotabweichung nach ventral.

**Table 1:** 

Zeitpunkt	Diagnose	Operation
Primäroperation	Abszedierende Spondylodiszitis LWK5/SWK1 bei Spondylolisthesis vera LWK5/SWK1 Meyerding-Typ II	1. Bandscheibenresektion und dorsale Spondylodese LWK5/SWK1
		2. Revision und Jet-Lavage 4 Tage nach Primäroperation
		3. Erneutes Debridement und Spongiosatransplantation von dorsal 14 Tage nach Primäroperation
3 Monate nach Primäroperation	Lockerung des Fixateur intern LWK5/SWK1 mit Einschmelzung LWK5	4. Verlängerungsspondylodese LWK4-SWK1 mit Schraubenwechsel
		5. Ventrale Spondylodese mit Beckenkammspan
14 Monate nach Primäroperation	Hypolordose und Fehlhaltung lumbosakral mit Pseudarthrose LWK5/SWK1 sowie ptotischem LWK5 versus SWK1	6. Dorsoventrodorsale Spondylodese LWK4-SWK1 mit Re-Spondylodese dorsal, ventraler Cage-Implantation LWK5/SWK1 sowie Nachreposition dorsal LWK5/SWK1
		7. Interposition eines Cages LWK4/5 mit ventraler Aufrichtung
5 Jahre nach Primäroperation	Implantatfehllage mit Pseudarthrose LWK4/5 und in Kyphose konsolidierter Olisthese LWK5/SWK1	8. Metallentfernung und Schraubenreinstrumentierung LWK4/5

*LWK* Lendenwirbelkörper, *SWK* Sakralwirbelkörper

## Befund

Radiologisch fanden sich hochpathologische Parameter für das sagittale Profil: So betrug die lumbale Lordose (LL) präoperativ nur 31°. Pelvic Tilt (PT) 37,1° und Sacral Slope (SS) 47,9° bei einer Pelvic Incindence von 85°, waren ebenfalls außerhalb der Norm. Das C7-Lot stand deutlich vor den Hüftköpfen (9,9 cm Abstand zur Hinterkante des SWK1).

## Diagnose

Chronische, therapieresistente Lumbofemoralgie bei sagittaler Imbalance durch 30° Defizit der LL bei Z. n. Fusion LWK4-SWK1.

## Therapie und Verlauf

**Figure 4:**
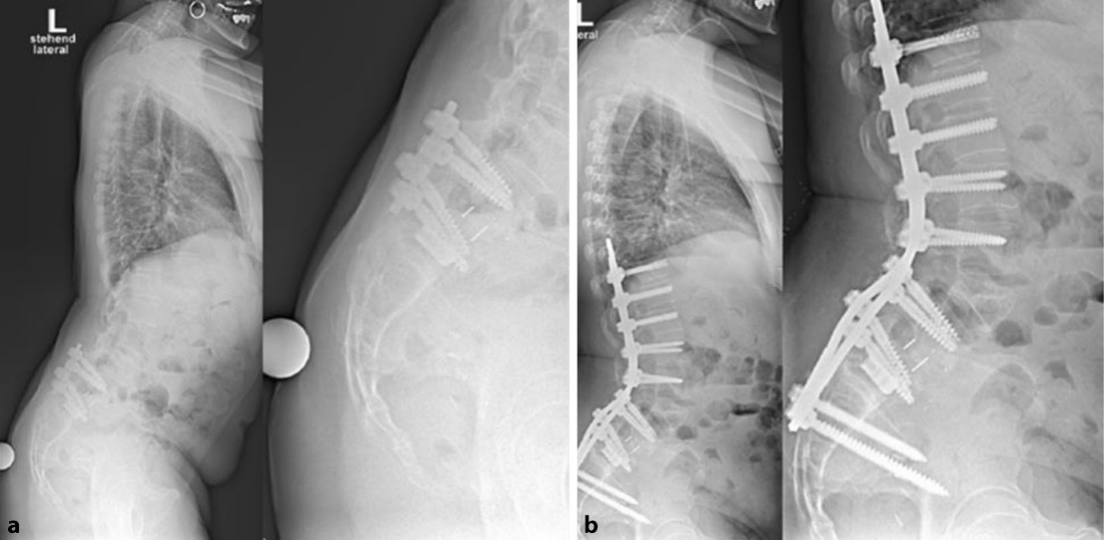

Wir stellten die Indikation zur dorsalen Verlängerungsspondylodese von BWK10 bis Ilium mit 95 mm SWK2-Ala-Iliumschrauben und Pedikelsubtraktionsosteotomie (PSO) LWK3 und führten diese komplikationslos unter Neuromonitoring durch (Abb. 4). Die Operationsdauer betrug 6 h mit einem Blutverlust von 1,5 l. Trotz intraoperativ festgestellter ausgeprägter Narbenbildung im Bereich der voroperierten Gebiete ist diese Operationsdauer im Einklang mit der bestehenden Literatur [1]. Die LL verbesserte sich in der postoperativen Ganzwirbelsäulenaufnahme auf 60° (+29°). PT und SS veränderten sich weniger deutlich auf 33,8° (−3,3°) bzw. 51,2° (+3,3°). Das C7–Lot verbesserte sich ebenfalls deutlich auf einen Abstand von 3 cm zur Hinterkante des SWK1.

**Figure 5:**
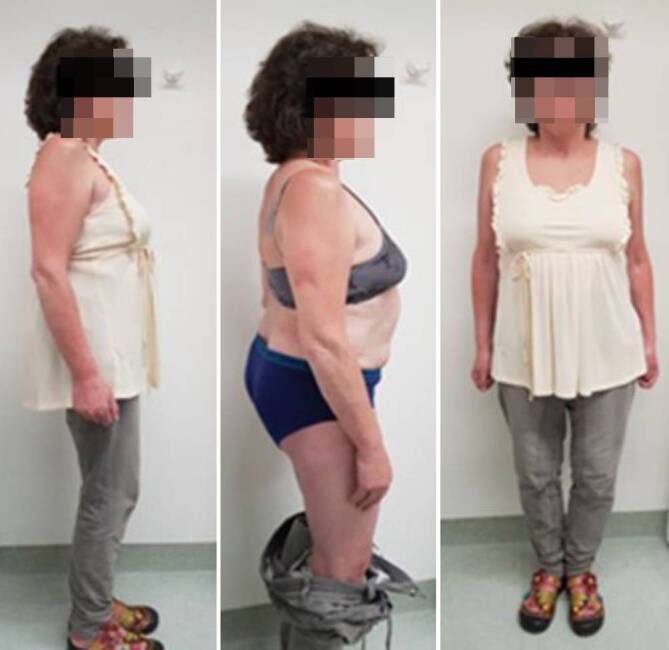

Im 3‑Monats-Follow-up berichtet die Patientin über eine vollständige Regredienz der Lumbofemoralgien und über eine exzellente Korrektur des sagittalen Profils (Abb. 5).

## Diskussion

Der aufgezeigte komplikative Verlauf der Patientin, welcher in einer langstreckigen Korrekturspondylodese endete, wirft nun verschiedene Fragen auf.

Die Indikation zum primären operativen Eingriff wurde aufgrund der diagnostizierten Spondylodiszitis LWK5/SWK1 bei vorbestehender Spondylolisthese gestellt. Zu diskutieren ist, ob ein Zwischenwirbelkörperinterponat die Einschmelzung des LWK5 mit lokaler Kyphosierung und damit die multiplen Folgeoperationen hätte verhindern können. So konnten Polly et al. nachweisen, dass eine Cage-Implantation die Steifigkeit des Konstrukts signifikant erhöht [7]. Weiter entlastet der Cage die eingebrachten Schrauben, was einer Schraubenlockerung entgegenwirkt und die LL verbessern kann [2, 5, 6].

Verschiedene Studien zeigen hierbei exzellente Ergebnisse bei Implantation von Interponaten [3, 8], allerdings keinen Unterschied zwischen Cages unterschiedlicher Größe (TLIF-, ALIF- oder PLIF-Cages) [9].

Alternativ kann jedoch auch körpereigener Knochen verwendet werden: Ein trikortikaler Beckenkammspan ohne Reposition der Olisthese gilt in einigen skandinavischen Zentren weiter als Goldstandard und zeigt auch bei der Spondylodiszitis gute Ergebnisse [3, 4]. Aus heutiger Sicht wäre neben einer sorgfältigen Ausräumung des Bandscheibenfachs bereits bei der initialen Operation ein Interponat indiziert gewesen. In unserer Klinik implantieren wir primär einen Titan-Cage mit möglichst großem Fußabdruck.

Die in diesem Fall erforderliche PSO wurde gewählt, um durch die möglichst kaudale Korrektur die entstandene Kyphosierung zu kompensieren und die harmonische Lordosierung der LWS, soweit möglich, wiederherzustellen. Eine Osteotomie auf Höhe des LWK4 war aufgrund von Narbenbildungen nach den Voroperationen nicht möglich. Alternativ hätte auch eine Cage-Implantation von LWK1 bis LWK4 diskutiert werden können. Allerdings wäre bei dieser Technik durch die Korrektur der lumbalen Lordose im kranialen Abschnitt der LWS laut Planung das C7-Lot weniger effektiv nach dorsal verlagert worden.

## Fazit für die Praxis

- Im Sonderfall einer kombinierten Spondylodiszitis mit Spondylolisthese sollte, wenn möglich, für die Verwendung eines Cages mit großem Fußabdruck plädiert werden.
- Besteht eine Störung des sagittalen Profils, so ist diese zu adressieren und mittels einer Cage-Implantation oder einer Osteotomie zu versorgen. Dabei sollte die Indikation zu Osteotomien der vorderen Säule (wie z. B. Pedikelsubtraktionsosteotomie) zurückhaltend gestellt werden.

## Abkürzungen

ALIF: „Anterior lumbar interbody fusion“
BWK: Brustwirbelkörper
LL: Lumbale Lordose
LWK: Lendenwirbelkörper
LWS: Lendenwirbelsäule
PLIF: „Posterior lumbar interbody fusion“
PSO: Pedikelsubtraktionsosteotomie
PT: Pelvic Tilt
SS: Sacral Slope
SWK: Sakralwirbelkörper
TLIF: „Transforaminal lumbar interbody fusion“
